# Identification of small GTPases as potential target proteins of the mycotoxin and renal carcinogen ochratoxin A

**DOI:** 10.1007/s00204-025-04189-8

**Published:** 2025-09-11

**Authors:** Johannes Borchers, Florinda Perugino, Andreas Schlosser, Stephanie Lamer, Leonie Lutz, Lorenzo Pedroni, Luca Dellafiora, Angela Mally

**Affiliations:** 1 Department of Toxicology, University of Würzburg, Versbacher Str. 9, 97078 Würzburg, Germany; 2 Rudolf Virchow Center, University of Würzburg, Würzburg, Germany; 3 Department of Food and Drug, University of Parma, Parma, Italy

**Keywords:** Ochratoxin A, Food contaminant, Carcinogenesis, Genotoxicity, Chemoproteomics, Docking, Molecular dynamics, Small GTPase

## Abstract

Ochratoxin A (OTA), a mycotoxin commonly found as a contaminant in a variety of foods, is known for its ability to cause kidney damage and tumors in rodents. Recent research indicates that replicative stress leading to aberrant mitoses and subsequent genetic instability may play a key role in OTA carcinogenicity. However, the specific molecular targets of OTA and early key events leading to replicative stress and mitotic disruption remain to be determined. In this study, a chemoproteomic workflow was employed to identify proteins that directly interact with OTA and its non-chlorinated analog ochratoxin B (OTB). To this end, OTA and OTB were immobilized on a stationary phase through covalent coupling to amine-functionalized agarose beads via their carboxy group. OTA-and OTB-functionalized beads were then incubated with kidney epithelial cell lysates to capture binding proteins for subsequent analysis via tandem mass spectrometry. Protein mass spectrometry identified several members of the family of small GTPases as specific OTA- and OTB-binding proteins. Moreover, a 3D molecular modeling approach integrating docking and molecular dynamics simulations was applied to study the mycotoxin–protein complex stability over time, providing mechanistic insights from an atomistic point of view. Ras superfamily GTPases, which were previously demonstrated to be transcriptionally deregulated in the presence of OTA, play crucial roles in various cellular functions, including DNA replication, mitosis, protein transport and cell adhesion, thus offering plausible links to cellular effects observed in response to OTA. In summary, results from this study for the first time identify small GTPases as direct molecular targets of OTA and suggest a potential role of small GTPases in OTA toxicity.

**Supplementary Information:**

The online version contains supplementary material available at 10.1007/s00204-025-04189-8.

## Introduction

Ochratoxin A (OTA) is a mycotoxin that occurs as a contaminant in a variety of foods, including cereal products, beer, coffee, spices, dried fruit, preserved meat, matured cheese, and milk. Since OTA is nephrotoxic and induces renal tumors in rodents, the presence of OTA in food presents a human health concern. Tumors in rats treated with OTA are thought to arise as a consequence of mitotic disruption and compensatory cell proliferation, leading to genetic instability and subsequent tumor formation (Adler et al. 2009; Czakai et al. 2011; Mally 2012; Rached et al. 2007). While recent in vitro studies point to unresolved replication stress as a cause for the mitotic defects and the associated spectrum of chromosomal damage (i.e., chromosome hypercondensation, abnormally separated chromatids, multipolar mitotic spindles, centrosome amplification, endoreduplication, polyploidy and aneuploidy) (Klotz et al. 2025; Schrenk et al. 2020), the primary molecular event that initiates the sequence of events leading to renal tumor formation by OTA remains to be identified. Evidence from in vitro and in vivo studies demonstrates that OTA does not form reactive metabolites with the potential to covalently bind to DNA and/or proteins (Delatour et al. 2008; Gautier et al. 2001; Gross-Steinmeyer et al. 2002; Mally et al. 2004; Zepnik et al. 2001). However, the potential of OTA to interact with distinct cellular proteins via high-affinity binding as a plausible molecular initiating event has not been investigated so far.

Chemoproteomics has emerged as a powerful approach widely used in drug discovery to investigate interactions between small molecules and proteins within complex biological systems, but, with the exception of profiling covalent protein adducts of reactive chemicals, is still rarely utilized in toxicological research to identify protein targets. While derivatization-free in-solution methods such as drug affinity responsive target stability (DARTS) (Lomenick et al. 2011), limited proteolysis coupled to mass spectrometry (LiP-MS) (Reber and Gstaiger 2023) or thermal proteome profiling (Franken et al. 2015) maintain the native ligand structure—thus avoiding chemical modification and steric hindrance that could alter binding properties—immobilization-based affinity chromatography (AC) remains among the most frequently employed techniques for target deconvolution (Chamrád et al. 2024; Terstappen et al. 2007). This technique relies on either covalent or reversible immobilization of the compound of interest to a stationary phase, incubation of the immobilized compound with a cell lysate, and subsequent identification of proteins bound to the immobilized compound via protein mass spectrometry.

**Fig. 1:**
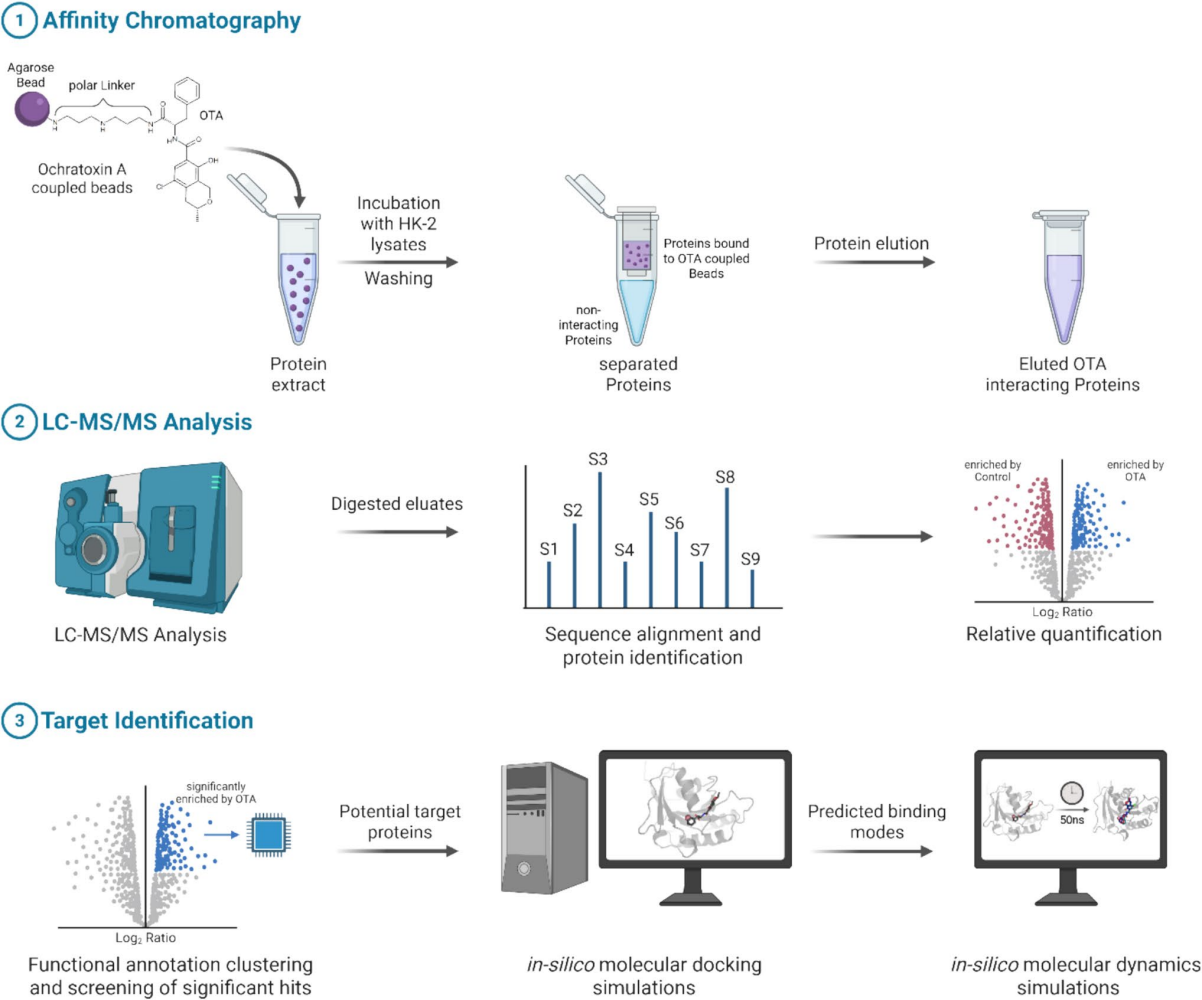
Experimental workflow, involving immobilization of OTA on agarose beads and affinity chromatography ①, identification of proteins bound to OTA coupled beads via LC–MS/MS ②, and subsequent in silico analyses ③. Figure created with BioRender

The overall objective of the present work was to provide further insights into the mechanism of OTA carcinogenicity by characterizing chemical–biological interactions to resolve the molecular initiating event involved in OTA carcinogenicity. Specifically, we utilized coupling of OTA to agarose beads via a polar linker and subsequent affinity chromatography chemoproteomics to identify putative target proteins of OTA and complemented this analysis by in silico target confirmation via docking and molecular dynamics simulations (Fig. 1).

## Materials and methods

### Chemoproteomics

#### Chemicals

Ochratoxin A (OTA) ≥ 98% (CAS No. 303-47-9, Cat. No. O1877) and *N*-(fluorenyl-9-methoxycarbonyl)-l-phenylalanine (Fmoc-Phe-OH, CAS No. 35661-40-6, Cat. No. 338338) were acquired from Sigma-Aldrich (Taufkirchen, Germany). Ochratoxin B (OTB) ≥ 98% (CAS No. 4825-86-9, Cat. No. 16167) was obtained from Cayman Chemical (Ellsworth MI, USA) (distributed by Biomol GmbH, Hamburg, Germany, Cat. No. Cay16167). EDC-HCl (CAS No. 25952-53-8, Cat. No. PG82079) was purchased from Thermo Fisher Scientific GmbH (Dreieich, Germany). Unless otherwise stated, all other standard reagents were from either Sigma-Aldrich (Taufkirchen, Germany) or Carl Roth GmbH (Karlsruhe, Germany).

#### Cell culture

The human proximal tubular cell line HK-2 (human kidney 2) was obtained from the American Type Culture Collection (Manassas, USA). Cells were cultured under standard cell culture conditions (37 °C, 5% CO_2_) in DMEM / F12 (1:1) (Sigma Aldrich, Taufkirchen, Germany) supplemented with 10% fetal bovine serum (FBS) (Thermo Fisher, Berlin, Germany), 2 mM l-glutamine (Thermo Fisher Scientific GmbH, Dreieich, Germany) and 1:100 penicillin/streptomycin (Merck, Darmstadt, Germany).

#### Preparation of affinity resins

OTA (5 µmol, 2.02 mg) was immobilized onto 2 mL 3,3’-diamino-dipropylamine (DADPA)-functionalized beaded agarose (CarboxyLink™ Coupling Resin, Thermo Scientific, Dreieich Germany) utilizing carbodiimide-mediated amide coupling chemistry (Supplementary Figure S1). To this end, 2 mL of agarose beads were equilibrated twice with 5 mL coupling buffer (0.1 M 2-(*N*-morpholino)ethanesulfonic acid (MES) pH 7.4, 150 mM NaCl). OTA (2.02 mg) was dissolved in 250 µL dimethylformamide (DMF), diluted with 1.75 ml coupling buffer, added to the equilibrated resin and incubated with end-over-end rotation for 10 min. 1-Ethyl-3-(3-dimethylaminopropyl)carbodiimide hydrochloride (EDC-HCl), 60 mg, ~ 62 eq.) was dissolved in 500 µL coupling buffer, immediately added to the equilibrated column and incubated with end-over-end rotation for 3 h at room temperature. The resin was then washed three times with 1 M NaCl (2 mL), equilibrated with 2 mL storage buffer (1 × PBS pH 7.4, 0.05% NaN_3_) and stored at 4 °C. Coupling efficiency was determined photometrically via OD_350nm_ of the reaction solution pre- and post-3 h incubation, with the latter also including wash fractions. Successful immobilization of OTA on the stationary phase was confirmed via visual inspection of OTA fluorescence of the resin after repeated washing under 366 nm UV light. For generation of OTB-functionalized beads, 1 mL of DADPA-agarose beads was coupled with 2.5 µmol (0.92 mg) OTB accordingly with respectively adjusted volumes and amounts (125 µL DMF, 875 µL coupling buffer, 30 mg EDC-HCl in 250 µL coupling buffer and 1 mL for each washing and equilibration step). To obtain l-phenylalanine-functionalized beads, 5 µmol (1.94 mg) fluorenylmethoxycarbonyl (Fmoc)-protected phenylalanine (Fmoc-l-Phe-OH) was immobilized onto 2 mL DADPA-agarose resin (Supplementary Figure S2). Coupling efficiency was determined photometrically via OD_257nm_ of reaction solution pre- and post-3 h incubation. Fmoc deprotection was performed by 3-min incubation with 3 mL 40% piperidine followed by 10 min incubation with 3 mL 20% piperidine. The resin was rinsed once with 20% piperidine and quantitative deprotection was confirmed photometrically based on the characteristic dibenzofulvene-piperidine adduct absorption at 301 nm of all piperidine fractions (Eissler et al. 2017). The resin was washed three times with 25, 50, and 25% DMF respectively, equilibrated with storage buffer and stored at 4 °C.

#### Preparation of cell lysates

*Whole cell lysate*: Pelleted cells from two ~ 75% confluent T-175 flasks (~ 35 × 10^6^ cells) were washed with cold 1 × PBS and re-suspended in 2 mL ice-cold lysis buffer (25 mM Tris–HCl pH 7.4, 150 mM NaCl, 1% Triton X-100, 2 mM EDTA, 5% glycerol) supplemented 1:100 with 20 µL Halt™ protease inhibitor cocktail (Cat. No.78430, Thermo Scientific™) and incubated on ice under constant agitation for 15 min. The lysis mix was then centrifuged for 20 min at 16,000 × g and 4 °C. The supernatant was placed into fresh tubes until further use while the pellet was discarded (final protein concentration ~ 5 mg/mL).

*Nuclear enriched extracts*: Pelleted cells from four ~ 75% confluent T-175 flasks (~ 70 × 10^6^ cells) were washed with cold 1 × PBS and re-suspended in 4 mL hypotonic lysis buffer (20 mM Tris–HCl pH 7.4, 10 mM NaCl, 3 mM MgCl_2_, 1 mM EDTA) supplemented 1:100 with protease inhibitor cocktail and left to swell for 15 min on ice under constant agitation. Triton X-100 was added to a final concentration of 0.5% and the cell suspension was vortexed at the highest intensity for 10 s. The suspension was centrifuged for 10 min at 720 × g and 4 °C. The supernatant was discarded, and the pellet was washed once with cold 1 × PBS. The pellet was re-suspended in 1 mL supplemented lysis buffer (see whole cell lysate) and incubated for 15 min on ice under constant agitation. The lysate was centrifuged for 20 min at 16,000 × g and 4 °C. The supernatant was placed into fresh tubes until further use, while the pellet was discarded (final nuclear extract protein concentration ~ 1.8 mg/mL).

Protein concentrations of samples were determined via the bicinchoninic acid (BCA) assay using the Pierce™ BCA Protein Assay Kit according to the manufacturer’s instructions.

#### Affinity chromatography / OTA target enrichment

Small molecule target enrichment was performed as recently described (Bach et al. 2017). 50 µL of OTA/phenylalanine/OTB coupled beads were washed twice with 500 μL of Pierce IP lysis buffer (Cat. No. 87787, Thermo). Equilibrated beads were incubated with freshly prepared and cleared HK-2 cell lysates for 3 h at 4 °C and overhead rotation. For whole cell lysates, 50 µL beads were incubated with 1 mL lysate (≙ ~ 5 mg protein) while nuclear enriched extract experiments were conducted using 0.5 mL (≙ ~ 900 µg protein) on 50 µL beads. Beads were briefly washed four times with 1 mL of cold wash buffer (20 mM HEPES pH 7.5, 115 mM NaCl, 1.2 mM CaCl_2_, 1.2 mM MgCl_2_, 2.4 mM K_2_HPO_4_, and 0.5% Triton X-100). Proteins were eluted with 400 μL of 1 × SDS sample buffer (60 mM Tris-HCl pH 6.8, 5% glycerol, 1.6% SDS, 100 mM DTT) at 70 °C for 10 min. Beads were spun down and supernatants were transferred into new vials for overnight protein precipitation at -20 °C after addition of 1.6 mL ice-cold acetone (fourfold sample volume). Pellets were washed thrice with acetone at -20 °C.

#### Reduction, alkylation, and gel electrophoresis

Precipitated proteins were dissolved in NuPAGE® LDS sample buffer (Invitrogen™), reduced with 50 mM dithiothreitol (DTT) at 70 °C for 10 min, and alkylated with 120 mM iodoacetamide at room temperature in the dark for 20 min. Separation was performed on NuPAGE® Novex® 4–12% bis–tris gels (Invitrogen™) with 3-(*N*-morpholino)propanesulfonic acid (MOPS) buffer (Invitrogen™, 50 mM MOPS, 50 mM Tris-Base, 0.1% SDS, 1 mM EDTA, pH 7.7, and 0.01–0.09% dimethylformamide) according to the manufacturer’s instructions. Gels were washed three times for 5 min with water and stained for 60 min with Simply Blue™ Safe Stain (Life Technologies). After washing with water for 1 h, each gel lane was cut into 15 slices.

#### In-gel digestion

The excised gel bands were destained with 30% acetonitrile in 0.1 M NH_4_HCO_3_ (pH 8), shrunk with 100% acetonitrile, and dried in a vacuum concentrator (Concentrator 5301, Eppendorf, Germany). Digests were performed with 0.1 µg trypsin per gel band overnight at 37 °C in 0.1 M NH_4_HCO_3_ (pH 8). After removing the supernatant, peptides were extracted from the gel slices with 5% formic acid, and extracted peptides were pooled with the supernatant.

#### NanoLC-MS/MS Analysis

NanoLC-MS/MS analysis was performed on an Orbitrap Fusion (Thermo Scientific) equipped with a PicoView Ion Source (New Objective) and coupled to an EASY-nLC 1000 (Thermo Scientific). Peptides were loaded onto a trapping column (2 cm × 150 µm ID, PepSep) and separated on a capillary column (30 cm × 150 µm ID, PepSep) both packed with 1.9 µm C18 ReproSil and separated with a 30-min linear gradient from 3 to 30% acetonitrile and 0.1% formic acid and a flow rate of 500 nl/min. Both MS and MS/MS scans were acquired in the Orbitrap analyzer with a resolution of 60,000 for MS scans and 30,000 for MS/MS scans. HCD fragmentation with 35% normalized collision energy was applied. A Top Speed data-dependent MS/MS method with a fixed cycle time of 3 s was used. Dynamic exclusion was applied with a repeat count of 1 and an exclusion duration of 30 s; singly charged precursors were excluded from selection. Minimum signal threshold for precursor selection was set to 50,000. Predictive AGC was used with AGC a target value of 4 × 10^5^ for MS scans and 5 × 10^4^ for MS/MS scans. EASY-IC was used for internal calibration.

#### MS data analysis

Raw MS data files were analyzed with MaxQuant version 1.6.2.2 (Cox and Mann 2008). Database search was performed with Andromeda integrated in MaxQuant. The search was performed against the UniProt Human Reference Proteome database (UP000005640). For OTA vs. Phe experiments, the database version used was from March 2021 (79,120 entries), while for OTB vs. Phe experiments, an updated version from November 2023 (82,685 entries) was used. Additionally, a database containing common contaminants was used to filter against contaminations. The search was performed with tryptic cleavage specificity with 3 miscleavages allowed. Protein identification was under control of the false discovery rate (FDR; < 1% FDR on protein and peptide spectrum match (PSM) level). In addition to MaxQuant default settings, the search was performed against the following variable modifications: Protein *N*-terminal acetylation, Gln to pyro-Glu formation (N-term. Gln) and oxidation (Met). Carbamidomethyl (Cys) was set as fixed modification. Further data analysis was performed using R scripts developed in-house. LFQ intensities were used for protein quantitation (Cox et al. 2014). Proteins with less than two razor/unique peptides were removed. Missing LFQ intensities were imputed with values close to the baseline. Data imputation was performed with values from a standard normal distribution with a mean of the 5% quantile of the combined log_10_-transformed LFQ intensities and a standard deviation of 0.1. For the identification of significantly enriched proteins, median log_2_ transformed protein ratios were calculated from the three replicate experiments and boxplot outliers were identified in intensity bins of at least 300 proteins. Log_2_ transformed protein ratios of sample versus control with values outside a 1.5x (significance 1) or 3x (significance 2) interquartile range (IQR), respectively, were considered as significantly enriched in the individual replicates.

### Filtering and functional annotation analysis

Proteins identified across all independent affinity enrichment experiments were filtered for significantly OTA (respectively OTB) enriched proteins by applying filters to significance = 2 (outside 3 × IQR) and Log_2_ Ratio > 0. Proteins had to be present in at least 2 of the 3 independent experiments. The generated lists of enriched proteins were submitted to DAVID https://davidbioinformatics.nih.gov/ (Huang et al. 2009; Sherman et al. 2022) as *Homo sapiens* gene lists and enrichment analysis performed against the *Homo sapiens* proteome as background. For generating gene ontology enrichment charts, only the GO term molecular function (GO MF direct) was considered using standard settings of medium stringency but a cutoff P value (EASE score) of 0.05 and a count threshold of 10. Annotation clustering considered GO MF direct as well as INTERPRO domain categories with standard settings and EASE score cutoff of 0.05.

### Molecular modeling studies

#### Data retrieval

The 3D structures of the small GTPases under analysis were retrieved from the Protein Data Bank (PDB, https://www.rcsb.org/) according to their good resolution and completeness of resolved sequence with the following PDB codes: 1N6O (Zhu et al. 2003) for Rab5a in GTP-bound state, 1TU4 (Zhu et al. 2003) for Rab5a in GDP-bound state, 1IBR (Vetter et al. 1999) for Ran in GTP-bound state, 3GJ0 (Partridge and Schwartz 2009) for Ran in GDP-bound state, 1A2B (Ihara et al. 1998) for RhoA in GTP-bound state, 1FTN (Wei et al. 1997) for RhoA in GDP-bound state, 2FN4 (10.2210/pdb2fn4/pdb) for Rras in GDP-bound state, 1OIW (Pasqualato et al. 2004) for Rab11a in GTP-bound state, 1OIX (Pasqualato et al. 2004) for Rab11a in GDP-bound state, 1T91 (Wu et al. 2005) for Rab7a in GTP-bound state and 8ZQ3 (Lu et al. 2024) for Rab7a in GDP-bound state. Due to the lack of completed crystallographic structures for Sar1A at the time of the analysis (May 2024), the 3D structure was derived from the AlphaFold (https://alphafold.ebi.ac.uk/) predicted structure retrieved in.pdb format from Uniprot (code: Q9NR31). Water molecules and all the co-crystallized ligands but the magnesium ion were removed from each structure before running the analysis. The wild-type structures of Rab5a and Rab7a in the GTP-bound state were obtained retro-mutating the Lys30 to Ala and Leu67 to Gln respectively (as per UniProt canonical sequences P20339 and P51149), using the Wizard Mutagenesis tool available in PyMol (version 2.5).

PubChem was used to retrieve 3D structures of OTA and OTα (CID: 442,530 and 107,911, respectively), while the crystallographic coordinates of ligands for the proteins under investigation were retrieved from PDB and used as positive controls (namely, GDP, GTP, GSP, and GNP).

#### Molecular docking

Docking simulations were performed to provide plausible architectures of binding for ligands under analysis. The software GOLD (Genetic Optimization for Ligand Docking, v. 2022.1) was used to perform docking analysis following a protocol keeping ligands fully flexible and proteins semi-flexible, allowing polar hydrogens free to rotate (Pedroni et al. 2022). The binding site was set at 10 Å radius sphere around the centroid of the respective co-crystallized ligand. For each complex under investigation, 10 poses were generated for each ligand and scored using the internal scoring function PLPScore (the higher the score, the more likely the binding architecture according to manufacturer declaration https://www.ccdc.cam.ac.uk).

#### Molecular dynamics simulations

Molecular dynamics (MD) simulations were performed using GROMACS (version 2022.6) (Abraham et al. 2015) to check the geometrical stability of protein–ligand complexes over time, in agreement with previous studies (Pedroni et al. 2022). The best scored docking poses obtained from docking analysis were used as input complexes for MD. All the ligands were parametrized with the SwissParam web server (https://ww.swissparam.ch) (Zoete et al. 2011), protein with GROMACS internal libraries, both using CHARMM27 all-atom force field (Brooks et al. 2009; Foloppe et al. 2000; MacKerell and Banavali 2000). First, each complex was solvated with SPCE water in a dodecahedron periodic boundary condition and neutralized adding Na^+^ or Cl^−^ as counter ions. Next, each system underwent an energetic minimization to both avoid steric clashes and correct improper geometries using the steepest algorithm with a maximum of 5000 steps. Before running MD simulations, each system underwent isothermal (300 K; coupling time of 2 ps) and isobaric (1 bar; coupling time of 2 ps) 100 ps simulations before running 50 ns long MD simulations (300 K with a coupling time of 0.1 ps and 1 bar with a coupling time of 2 ps).

## Results and discussion

### OTA target identification via chemoproteomics

#### Affinity resin functionalization

Identification of OTA target proteins requires immobilization of the OTA molecule onto a stationary phase. OTA provides two functional groups that are accessible for derivatization and immobilization, i.e., the carboxylic acid function of the phenylalanine moiety and the phenolic hydroxyl group of the isocoumarin motif. Although the phenylalanine moiety is known to contribute to OTA toxicity in vivo predominantly by affecting OTA toxicokinetics via facilitating binding of OTA to plasma proteins (Perry et al. 2004), there is evidence to suggest that it is not a structural requirement for biological effects induced by OTA in vitro. Several studies demonstrate that ochratoxin α (OTα), a metabolite resulting from cleavage of the amide bond and thus lacking the phenylalanine moiety, causes similar cyto- and genotoxic effects, such as aberrant mitotic progression and micronuclei formation, as OTA in vitro (Czakai et al. 2011; Miguel Alfonso et al. 2022; Steyn et al. 1975). Therefore, we considered the phenylalanine carboxyl group as the most appropriate functional site to link OTA to the stationary phase. In addition to OTA, we also included its non-chlorinated analog OTB in our analysis. While OTB is considered less toxic in vivo compared to OTA due to faster elimination (Mally et al. 2005), it exhibits similar cytotoxicity and induces the same mitotic aberrations as OTA in vitro (Czakai et al. 2011; Mally et al. 2005), suggesting that it shares the same mechanism and primary molecular targets.

**Fig. 2:**
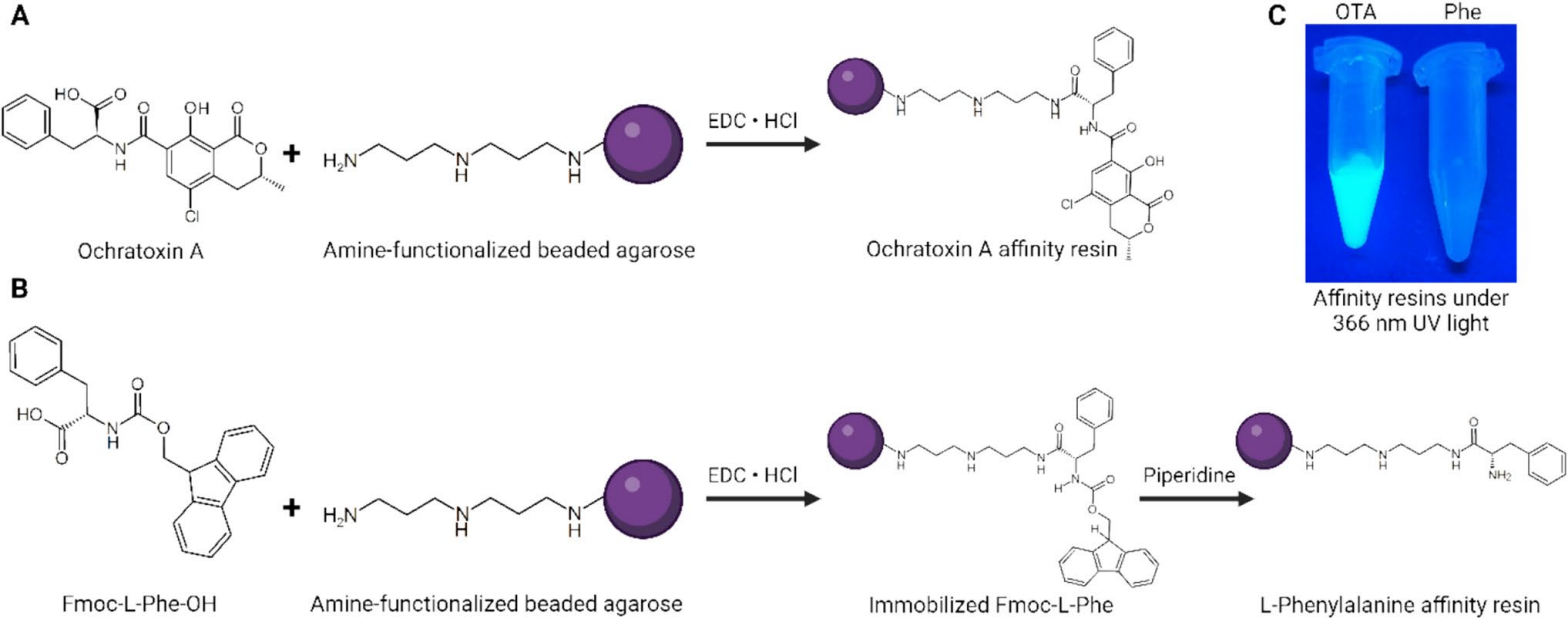
Scheme showing affinity resin functionalization: **A** Water-soluble carbodiimide-mediated peptide coupling of ochratoxin A (OTA) to DADPA functionalized agarose beads. **B** Analogous coupling of Fmoc-protected l-phenylalanine and deprotection using piperidine. **C** Washed OTA- and Phe-functionalized agarose resins after coupling (and deprotection) reactions under 366 nm UV light, showing the characteristic fluorescence of OTA coupled to the matrix. Figure created with BioRender

Carboxylic acids can be easily coupled covalently to amine functions using carbodiimides such as EDC to activate the carboxy function analogous to solid-phase peptide synthesis chemistry. Therefore, OTA was coupled to diamino-dipropylamine (DADPA)-functionalized beaded agarose (CarboxyLink™, Thermo Scientific™) via the polar DADPA spacer (Fig. 2A). l-Phenylalanine (Phe) was selected as a structurally related, yet inactive small molecule to serve as a control ligand and was thus immobilized onto amine-functionalized agarose beads (Fig. 2B). To this end, the free amino group of phenylalanine was protected by a fluorenylmethoxycarbonyl (Fmoc) group prior to coupling with amine-functionalized agarose beads, and subsequently deprotected using piperidine.

Successful coupling was confirmed based on the absorption of the reaction mix prior and after coupling / deprotection reactions as well as by monitoring OTA/OTB fluorescence under UV light (Fig. 2C). Coupling efficiency was determined to be > 90% based on photometric measurements of the reaction mix pre- (OD_350_ = 1.126) and post-incubation (OD_350_ = 0.108, including wash fractions and accounting for dilution) and OTA functionalization was accordingly estimated at around ~ 2.25 µmol/mL bead volume.

#### Affinity chromatography coupled with quantitative label-free mass spectrometry identify small GTPases as potential OTA targets

To identify potential molecular targets of OTA (and its non-chlorinated analog OTB), OTA- and OTB-binding proteins were captured from human proximal tubule epithelial cells (HK-2) whole cell lysates and nuclear enriched fractions by affinity chromatography using the OTA- and OTB-functionalized affinity resins and were subsequently analyzed by quantitative label-free mass spectrometry. To further exclude and limit unspecific binding proteins from analysis, affinity enrichment was performed against phenylalanine-functionalized affinity resins (Phe-functionalized resins). Considering that OTA genotoxicity is most likely linked to DNA associated nuclear processes, affinity capture experiments were performed from nuclear enriched fractions in addition to whole cell lysates of human proximal tubule epithelial cells (HK-2). This sub-fractionation was done to increase proteome coverage but also to limit competitive displacement of specific binding but low abundance proteins by large and high abundance cytoplasmic proteins. After incubation with respective lysates, OTA-, OTB-, and Phe-functionalized resins were washed and the remaining proteins bound to OTA, OTB, or Phe were eluted, reduced, alkylated, and digested before LC–MS/MS analysis.

**Fig. 3:**
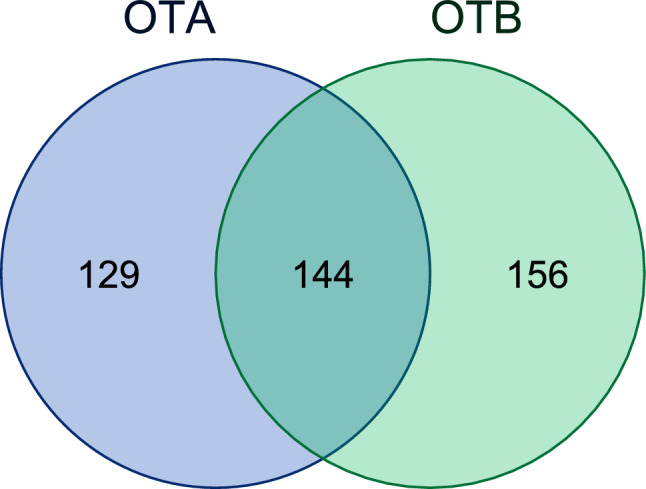
Venn diagram showing the overlap between putative target proteins of OTA and OTB. This overlap of 144 proteins is likely to underrepresent the true similarity, as isoform-specific variants of a protein (e.g., RAP2C vs. RAP2B) are listed as individual proteins

Intensities of all identified proteins were blotted against intensity ratios (e.g., OTA vs. Phe) determined by label-free quantification. In each independent experiment, on average, ~ 4200 proteins were identified (Supplementary Table 1) and data obtained from three independent experiments were subsequently filtered for proteins specifically enriched by OTA (respectively OTB) against Phe (Significance 2, Log_2_ Ratio > 0). Based on these criteria, 273 proteins significantly enriched from nuclear extracts by OTA, thus representing putative OTA target proteins, were identified (Supplementary Table 1). For OTB, 300 proteins were significantly enriched and thus identified as putative OTB target proteins. Of these significantly enriched proteins, 144 were shared between OTA and OTB (Fig. 3).

**Fig. 4:**
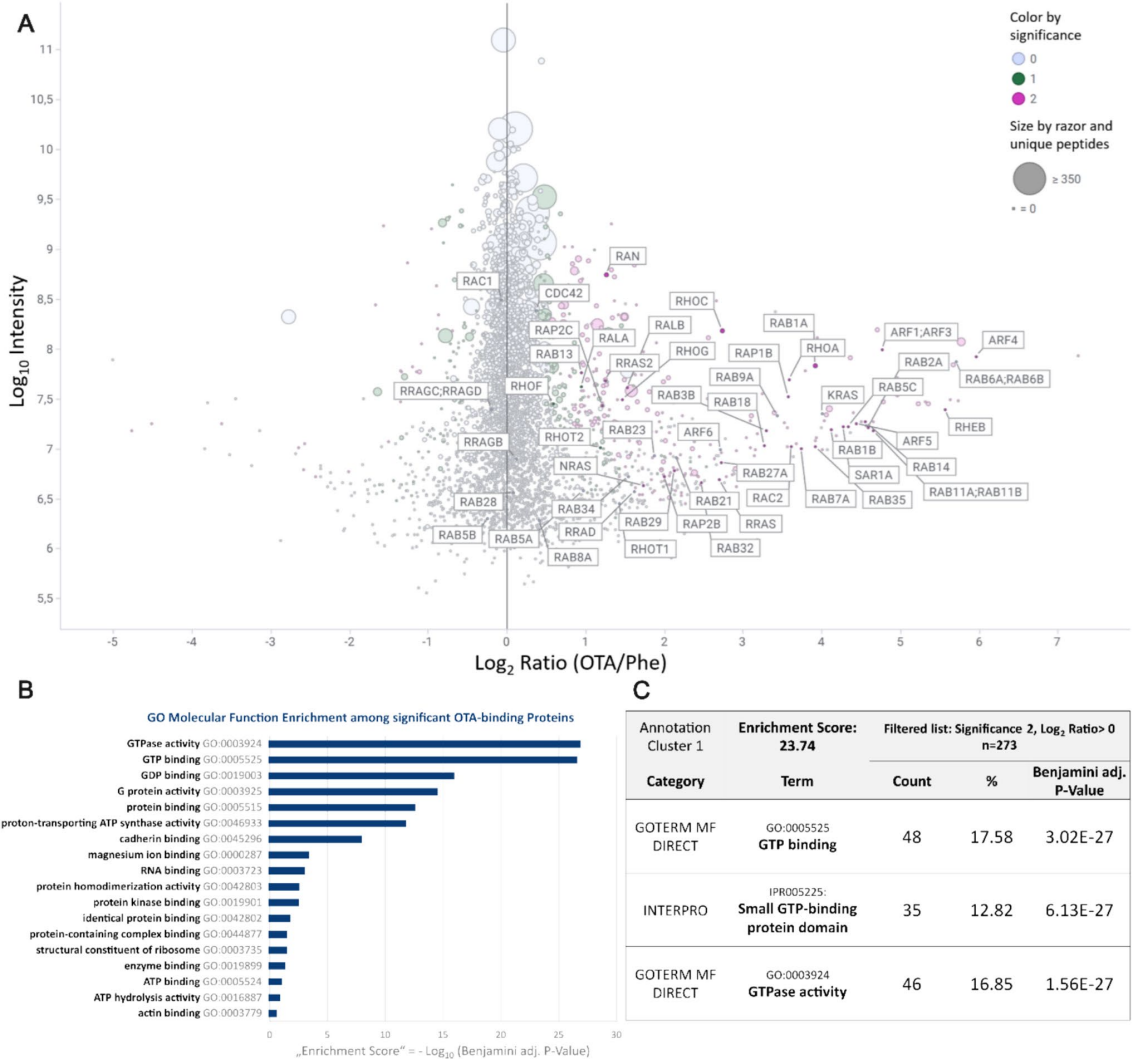
OTA target protein identification using OTA vs. Phe affinity chromatography of nuclear enriched HK-2 extracts. **A** Relative quantification of all identified proteins with intensities and intensity ratios presented as median of 3 independent biological replicates. Proteins labeled with their gene name represent Ras superfamily proteins, showing their high prevalence among the proteins significantly enriched by OTA vs. Phe affinity chromatography (positive Log_2_ ratio, purple). **B** Molecular function gene ontology analysis showing significant enrichment of GTPase functionality, G protein activity and related terms. **C** Functional annotation clustering of molecular function and InterPro category terms revealed GTPases, mostly consisting of Ras superfamily small GTPases, as functional annotation cluster with the highest enrichment score (-Log_10_ transformed mean of *P* values of contained enriched terms) and fold enrichment (Color figure online)

Relative quantification of proteins identified in the OTA vs Phe capture experiments using nuclear enriched HK-2 fractions is shown in Fig. 4A, with intensities and intensity ratios presented as median of 3 independent experiments. The set of significantly enriched proteins comprises proteins with diverse molecular functions (e.g., RNA, actin or ribosome binding and oxidoreductase, aminotransferase, or kinase activity) involved in various biological processes (e.g., protein transport, translation initiation, migration, tRNA splicing, or iron transport), with no immediately obvious link to OTA genotoxicity. Gene ontology (GO) enrichment analysis using DAVID revealed GTP-binding proteins with GTPase and G protein activity as a highly enriched subgroup of OTA-interacting proteins with the highest enrichment scores and among the highest fold enrichments (Fig. 4B). Annotation clustering of GO molecular function and InterPro category terms (Fig. 4C) additionally highlighted ~ 73% of these GTP-binding proteins as members of the Ras Superfamily of small GTPases (Fig. 4A), which includes five subfamilies, i.e., Ras, Rho, Rab, Sar1/Arf, and Ran. Representatives of all subfamilies were identified among the significantly OTA-enriched GTP-binding proteins (Table 1), whereby the majority of smallGTPases identified as putative OTA-binding proteins belong to the Rab and Ras subfamilies.

**Table 1:**
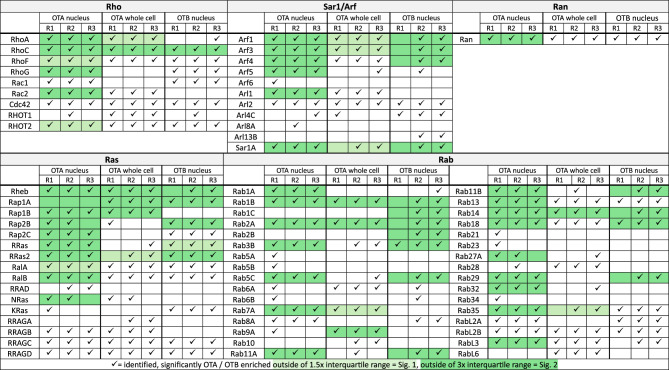
Overview of Ras superfamily small GTPases identified as putative OTA/OTB target proteins by affinity chromatography-based chemoproteomics, sorted by subfamily (Color figure online)

Small GTPases identified in each individual experiment are marked by a checkmark (✓). Small GTPases significantly enriched by OTA/OTB across all independent experiments (*n* = 3) are highlighted by significance level (light green: significant, Sig = 1, green: very significant, Sig = 2)

**Fig. 5:**
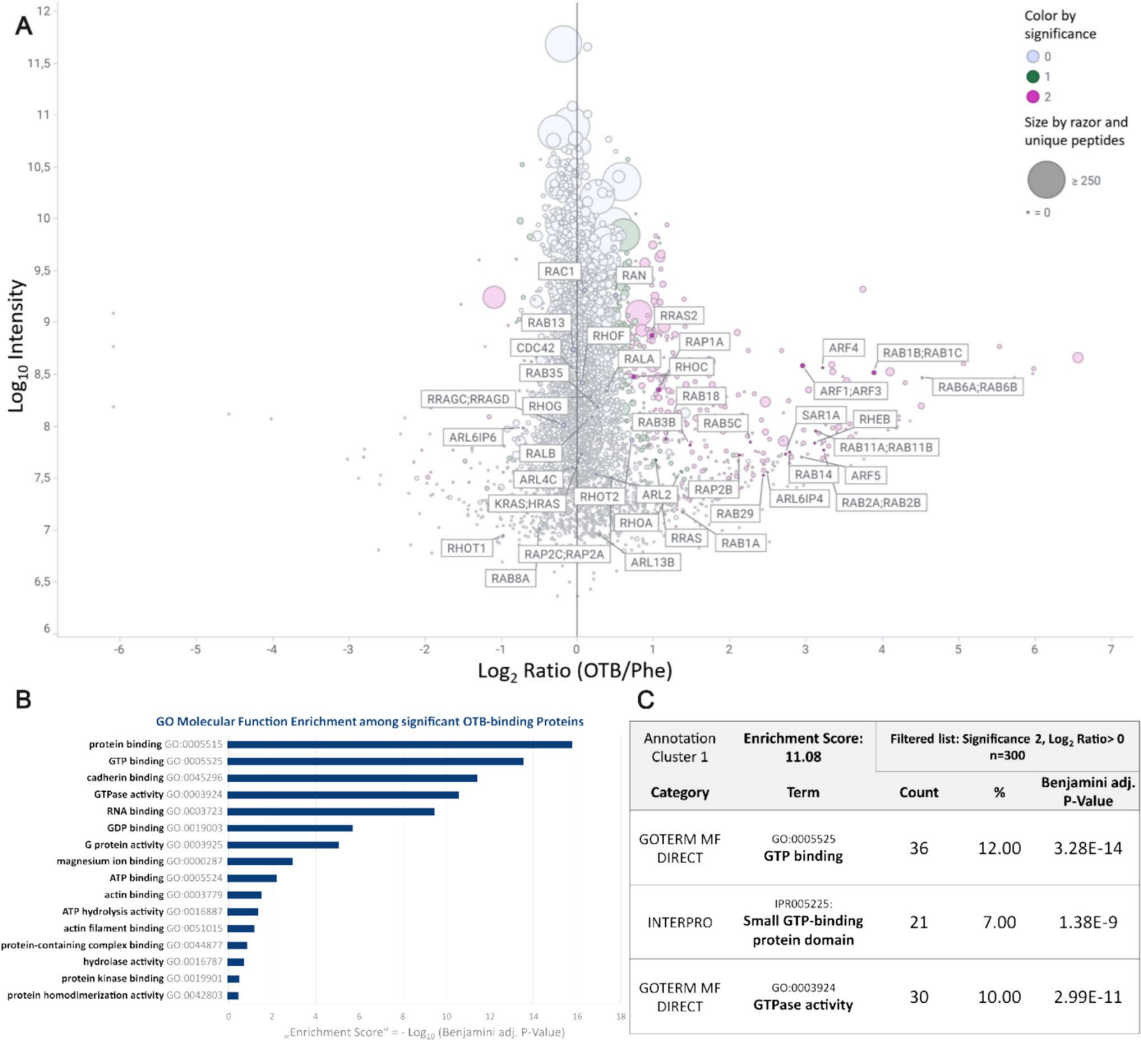
OTB target protein identification using OTB vs. Phe affinity chromatography from nuclear enriched HK-2 extracts. **A** relative quantification of all identified proteins with intensities and intensity ratios being median values of 3 independent biological replicates. Labeled proteins represent Ras superfamily proteins, showing their prevalence among the proteins significantly enriched by OTB vs. Phe affinity chromatography (positive Log_2_ ratio, purple). **B** Molecular function gene ontology analysis showing significant enrichment of GTPase functionality, G protein activity and related terms. **C** Functional annotation clustering of molecular function and InterPro category terms revealed GTPases, mostly consisting of Ras superfamily small GTPases, as functional annotation cluster with the highest enrichment score (-Log_10_ transformed mean of P values of contained enriched terms) and fold enrichment

Functional annotation analysis of OTA-binding proteins identified from whole cell extracts (Supplementary Fig. 3) and proteins significantly enriched by OTB vs. Phe affinity chromatography (Fig. 5) also revealed GTP-binding, small GTP-binding protein domain and GTPase activity as the most significantly enriched annotation terms. Compared to results obtained from OTA affinity chromatography experiments conducted with nuclear HK-2 cell extracts, enrichment scores of GTPase-related GO terms obtained in whole cell extracts were less significant and fewer small GTPases reached significance in enrichment, which may be explained by competitive binding of abundant cytosolic proteins in the lysate. Similarly, functional enrichment of small GTPases in the OTB captured proteins showed lower statistical significance enrichment compared to OTA. This was primarily influenced by the first replicate, which deviated to some extent from the other two replicates (Table 1, Supplementary Fig. 4). Overall, however, these data support small GTPases as specific ochratoxin interacting proteins.

**Fig. 6:**
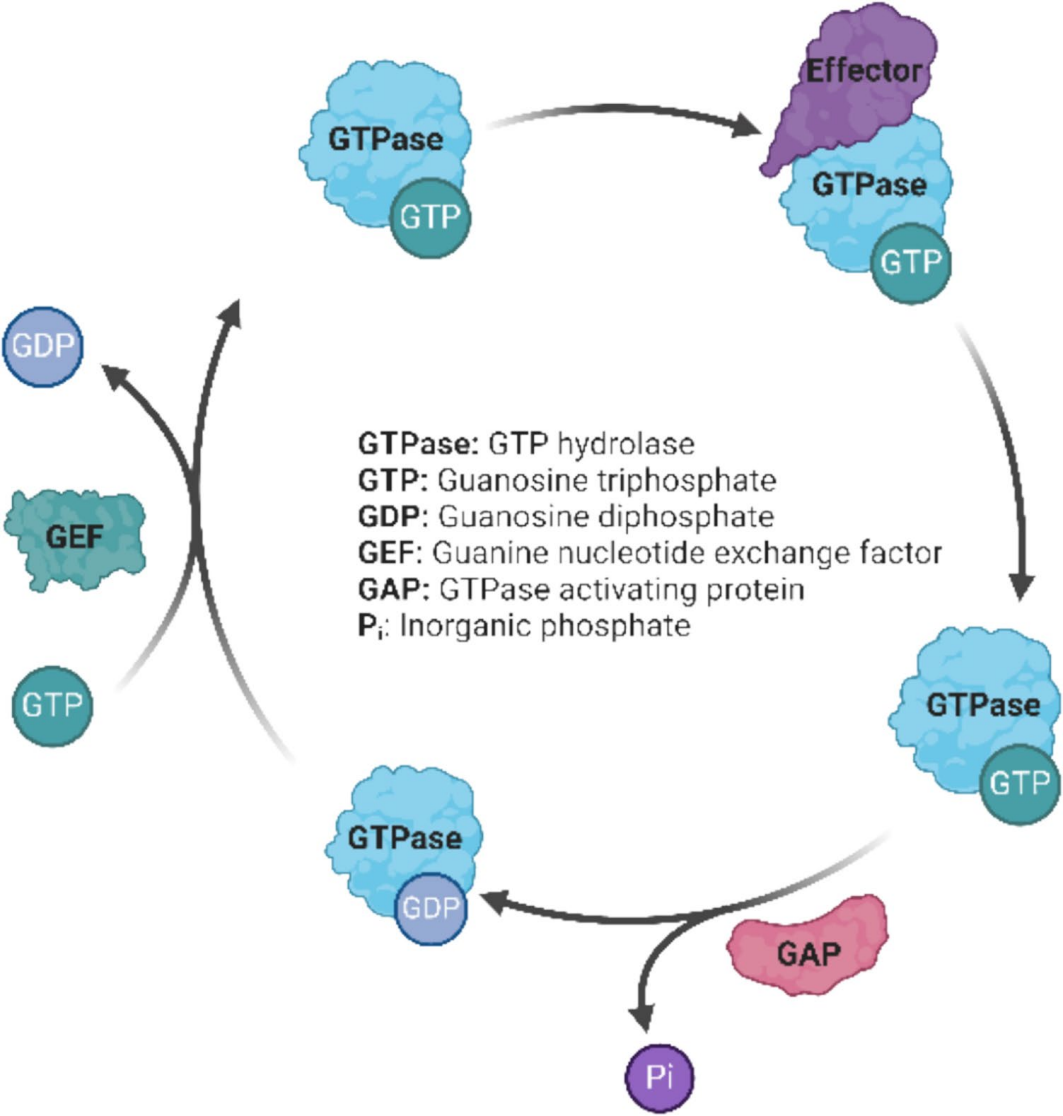
Simplified schematic illustration of the functional cycle of GTPases / G proteins: In its active state, the GTPase is bound to GTP, promoting downstream signaling by binding effector proteins. GTPase-activating proteins (GAPs) facilitate the hydrolysis of GTP to GDP, converting the GTPase to its inactive GDP-conformation state. Guanine nucleotide exchange factors (GEFs) facilitate the exchange of GDP for GTP, reactivating the GTPase. Figure created with BioRender

Small GTPases, also known as small G proteins, represent one of the two groups of G proteins that act as important molecular switches in various cell signaling pathways (Forbes et al. 2022). Unlike their related heterotrimeric “large” G proteins associated with G protein-coupled receptors (GPCRs), they act as monomeric switches that do not rely on receptor mediated activation (Berg et al. 2023). As G protein switches, they cycle between an GDP-bound “inactive” and a GTP-bound “active” state (Fig. 6). GTPase activity of these hydrolases is very low and GTP hydrolysis usually only occurs upon binding of a GTPase-activating protein (GAP), which returns the GTPase in its inactive GDP-bound state (Mishra and Lambright 2016). A guanine nucleotide exchange factor (GEF) then facilitates exchange of GDP with fresh GTP to activate the GTPase again (Toma-Fukai and Shimizu 2019). Conformational Change between inactive and active state determines affinity toward effector proteins, which sometimes also act as respective GAPs. GEF and GAP activity in turn is typically regulated by other signaling processes from both internal and external stimuli. The small GTPase subfamilies share 5 conserved G domains, which determine the base GTPase functionality (Song et al. 2019). The variable non-conserved domains define effector affinities and thus biological process specificity. Considering that representatives of all subfamilies involved in regulation of a variety of cellular processes were identified as OTA-interacting proteins, binding of OTA to conserved G domain sites such as the GDP-/GTP-binding pocket or switch 1 or 2 regions seems plausible. In order to support direct molecular interaction of OTA with GTPases and infer potential effects of OTA, in silico docking and molecular dynamics simulations were used to investigate the interaction of OTA and its metabolite OTα with representatives of each subfamily of small GTPases identified as putative molecular targets of OTA.

### Molecular modeling

**Fig. 7:**
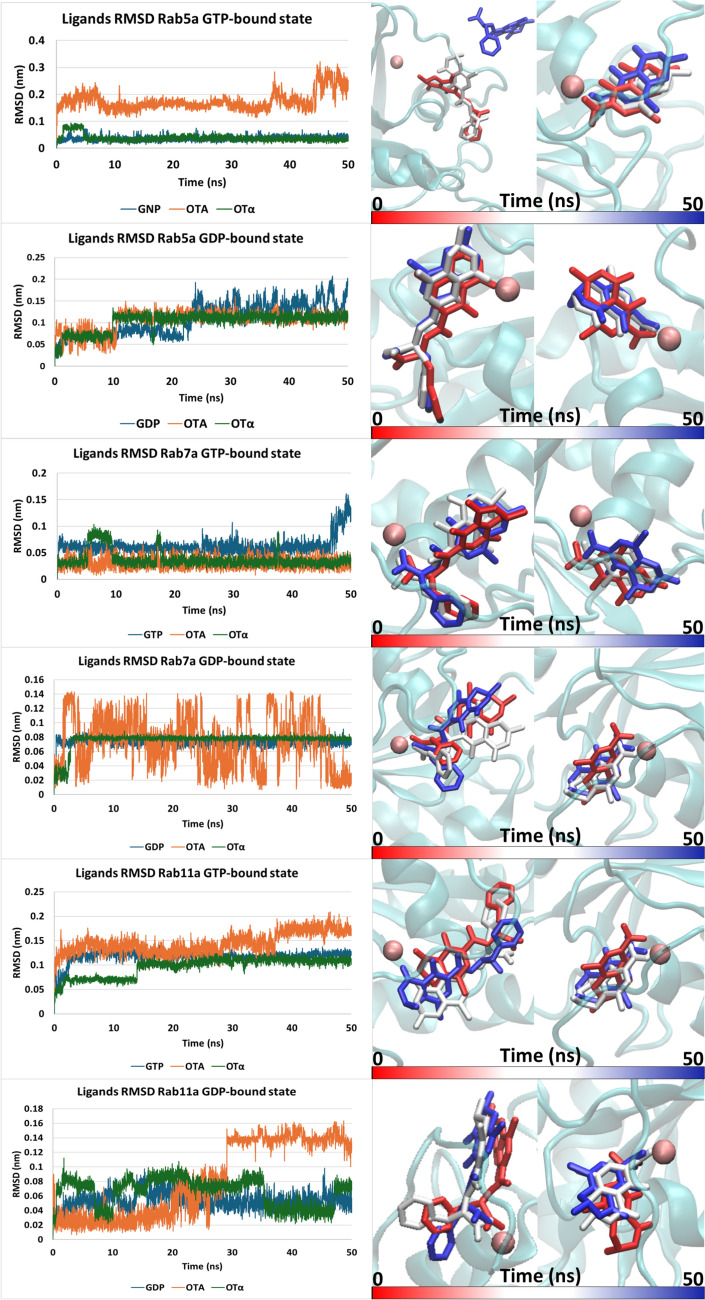
On the left, RMSD plots of co-crystallized ligands (blue), OTA (orange) and OTα (green) in complex with the Rab GTPases under investigation. On the right, time-step representation of OTA (left) and OTα (right). The from-red-to-blue color switch indicates the stepwise changes of the ligands’ coordinates along the simulation (50 ns) (Color figure online)

As most of the small GTPases identified as putative OTA targets via chemoproteomics belong to the Rab family, which also represents the largest of the Ras superfamily, three representative Rab family members were selected for docking and subsequent MD simulations. For the remaining GTPase families, a representative member identified as a putative OTA target protein was selected. In addition to modeling the molecular interaction of OTA with small GTPases, we were also interested to investigate the capability of ochratoxin α (OTα), a major OTA metabolite that results from cleavage of the peptide bond, to interact with the selected GTPases (Zepnik et al. 2003). Docking analysis predicted the binding pose of both OTA and OTα within the nucleotide-binding site of small GTPases under investigation. All the GTPases–OTA and GTPases–OTα complexes recorded a positive docking score (the higher the score, the better the interaction; Table 2), highlighting the theoretical capability of OTA and its metabolite to satisfy the pharmacophoric requirement of the respective pocket. According to results shown in Table 2, OTα generally exhibits PLPScores lower than those recorded by OTA and the co-crystallized ligands. However, it is important to note that docking scores, including GOLD PLPScore, might be affected by the size of the molecules. Therefore, docking analysis was integrated with molecular dynamics simulations to refine predictions, in agreement with previous studies (Louisse et al. 2023, 2022). In most of the OTA–GTPase complexes, the architecture of binding revealed the interaction between the carboxyl group of the OTA phenylalanine moiety and the Mg^2+^ ion, which is required for nucleotide-binding. While the carboxyl group of the phenylalanine moiety was employed for immobilization of OTA on the stationary phase and was therefore likely masked in our chemoproteomics approach, it is possible that coordination between the Mg^2+^ ion and oxygen containing functional groups in close vicinity to the phenylalanine carboxyl group may have contributed to binding of OTA to small GTPases. Conversely, Rab5a both in the GTP and GDP-bound state and Rab11a in the GTP-bound state (Fig. 7) showed the interaction between the isocoumarin moiety of OTA and the Mg^2+^ ion. In contrast, arrangement of OTα was consistent across all GTPases investigated regardless of the GTP-/GDP-bound state. Here, the carboxylic group of OTα arranged close to the Mg^2+^ ion at the small GTPase-binding site, reasonably entering its coordinating space and contributing to the energy of binding. Moreover, for the sake of validation and comparison, all the natural ligands co-crystallized in the structures under investigation were calculated as positive controls.

**Table 2:** Molecular docking scores (PLPScore) of OTA, OTα, and co-crystallized ligands (taken as positive controls) within the GTPases under investigation

GTPases	OTA	OTα	Co-crystalized ligand	
Rab5a GTP	89.31	76.91	158.05	
Rab5a GDP	70.03	61.43	103.22	
Rab7a GTP	92.01	60.63	122.91	
Rab7a GDP	73.91	58.25	87.66	
Rab11a GTP	96.19	64.06	127.05	
Rab11a GDP	86.1	59.87	123.87	
Ran GTP	65.54	49.05	123.37	
Ran GDP	68.82	60.55	117.73	
RhoA GTP	86.33	75.21	142.3	
RhoA GDP	87.04	63.64	108.7	
Rras GDP	86.06	66	123.92	
Sar1A GDP	74.37	61.83	109.75	

The higher the score, the better the physico-chemical match with the pocket (as per manufacturer declaration (https://www.ccdc.cam.ac.uk)

Overall, the outcomes collected from our docking studies represented first evidence of the capability of OTA and OTα to bind the nucleotide-binding pocket of small GTPases, potentially resulting in their dysregulation. However, based on previous evidence showing that MD simulations may refine docking outcomes, thereby improving predictions (Louisse et al. 2022), MD simulations were performed to assess the complexes´ stability over time. For each complex, the best scored docking pose was used as input for the MD simulations. The protein–ligand stability was investigated by analyzing ligand trajectories and the geometrical stability, calculating the root mean square deviation (RMSD) in agreement with previous studies (Pedroni et al. 2023). More in detail, ligand trajectory RMSD evaluation is a key dissection to determine if a ligand remains stably bound or detaches from the binding site, where a steady trend with low variability typically reflects geometrically stable interaction and appreciable binding. Analysis of RMSD and trajectories revealed that OTA can interact quite stably either via the Phe- or isocoumarin moiety, depending on the system under investigation, whereby the oxygen containing functional groups coordinate with Mg^2+^ of the binding pocket, which is required for nucleotide-binding.

*Rab family.* As shown in Fig. 7, among the investigated Rab members (i.e., Rab5a, Rab7a and Rab11a), which are all linked to the endosome pathway, OTA showed stability in complex with Rab5a in its GDP-bound state, while detached when in complex with Rab5a in the GTP-bound state. This behavior is highlighted in the first panel of Fig. 7 retracing the detachment of OTA during the simulation. Conversely, when in complex with Rab7a and Rab11a, OTA could interact with the protein regardless of the GTP-/GDP-bound state although RMSD plot analysis (the steadier over time, the better the interaction) and trajectories inspection revealed a more stable interaction with GTP-bound proteins. Concerning OTα, a stable interaction within the Rab family GTPases under investigation was observed in both GTP- and GDP-bound states.

The crystallographic ligands GTP, GSP, and GDP were used as positive controls. Their MD outcomes were compared to those of OTA and Otα, revealing a RMSD trend substantially comparable to that observed with OTα across all investigated Rab GTPases irrespective of the GDP-/GTP-bound state, as well as with OTA in the Rab5a GDP-bound state, Rab7a GTP-bound state and Rab11 GTP-bound state.

**Fig. 8:**
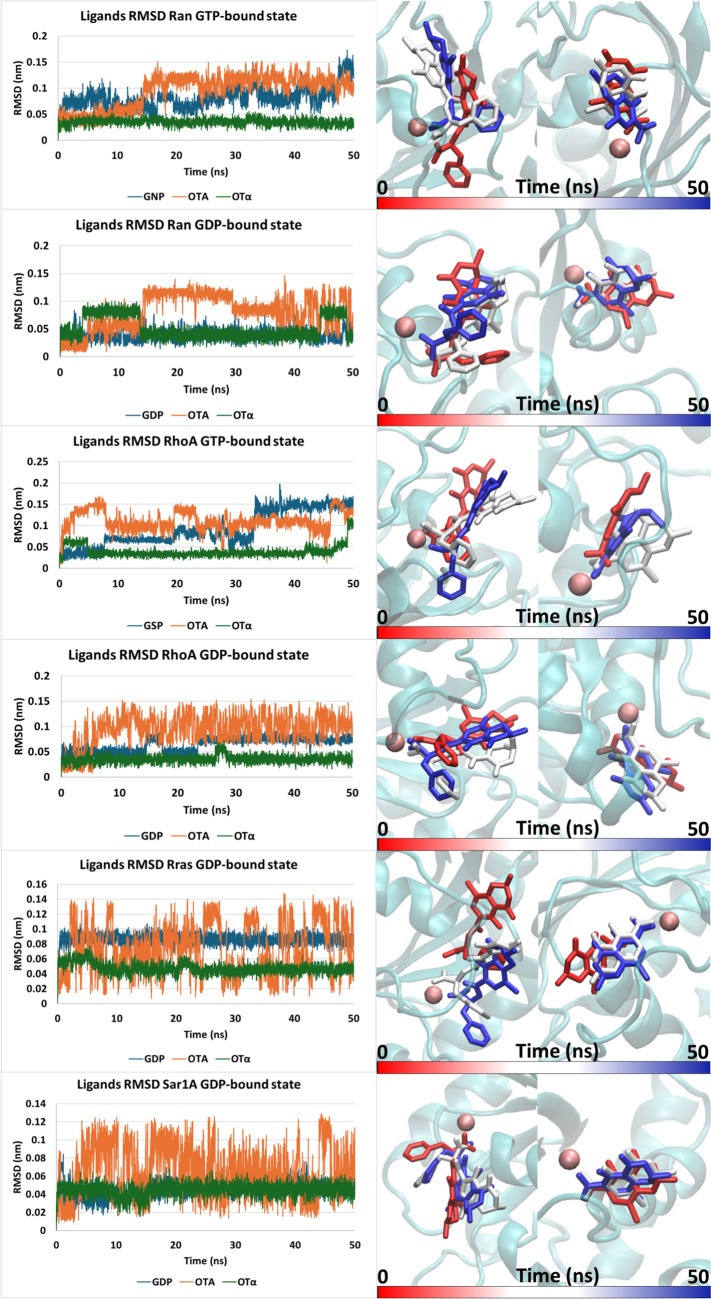
On the left, RMSD plots of co-crystallized ligands (blue), OTA (orange) and OTα (green) in complex with the Ran, RhoA, Rras and Sar1A GTPases under investigation. On the right, time-step representation of OTA (left) and OTα (right). The from-red-to-blue color switch indicates the stepwise changes of the ligands’ coordinates along the simulation (50 ns)

*Rho family.* As a representative of the Rho family, RhoA was studied in the GDP and GTP conformation (Fig. 8). The crystallographic ligands GDP and GSP were used as positive control for the two states, respectively, and their MD outcomes were compared to those of OTA and OTα. Concerning OTα, it showed with both RhoA-bound states, an RMSD trend even lower in terms of nm fluctuations than that observed for the respective crystallographic ligands, pointing to the geometrical stability of its interaction over time. Regarding OTA, RMSD analysis revealed a trend comparable to GSP in the RhoA GTP-bound state. On the other hand, a lower geometrical stability was recorded for OTA when in complex with RhoA in GDP-bound state, particularly compared to the Rab5a GDP-bound state, Rab7 GTP-bound state and Rab11 GTP-bound state complexes. This suggests that OTA may be a suboptimal ligand of RhoA in the GDP-bound state.

*Ras family.* Rras was selected as a representative of the Ras family. However, at the time of the analysis, the only structure available for this enzyme was in the GDP-bound state. Based on RMSD plot and trajectories inspection (Fig. 8), MD outcomes revealed a higher capability of OTα compared to OTA to interact with the nucleotide-binding site. Specifically, OTα showed a steady interaction suggesting a geometrically stable binding, while OTA recorded an RMSD plot with large variations that underline its geometric instability as confirmed by the inspection of the trajectory. The crystallographic ligand GDP, investigated as a positive control, revealed that the RMSD trend observed for OTα was lower than that recorded for GDP, further confirming the stability of the interaction calculated for OTα.

*Ran family.* The only member analyzed belonging to the Ran family was the Ran protein. Both the GDP- and the GTP-bound states were investigated. The crystallographic ligands GDP and GNP were analyzed as positive controls. RMSD analysis revealed a lower trend for OTα compared to the crystallographic ligand (i.e., GNP) in the Ran GTP-bound state and a trend comparable to GDP in the Ran GDP-bound state. OTA showed an RMSD trend comparable to GNP and higher than GDP, but still in a low nm range. These findings indicate considerable stability of OTα but suboptimal capability of OTA to interact with both the conformations.

*Arf family.* Sar1A was studied among the members of the Arf family. Since all structures available on PDB at the time of the investigation were incomplete, the AlphaFold structure was chosen. The natural ligand (GDP) was analyzed as positive control. The time-step trajectories and the RMSD analysis revealed that both OTA and OTα can interact with this small GTPase (Fig. 8). OTα showed an RMSD trend comparable to that of GDP, while OTA exhibited a higher and apparently rather variable trend, which occurred in a very low nm range (Fig. 8). These results suggest that the interaction of OTA with Sar1A is not geometrically very stable, whereas the complex with OTα appears to be stable.

Overall, these findings indicate that both OTA and OTα may interact with most of the small GTPases under investigation, with OTα showing an overall higher stability when in complex with small GTPases compared to OTA and thus potentially representing a stronger inhibitor. In light of the insights discussed above, the first GTPases deserving further investigations and a potential follow-up work may be Rab5a GDP-bound state and Rab7a in GTP-bound state being the two small GTPases which appear to stably interact with both OTA and OTα. It is interesting to note that the capability of OTA and OTα to interact with small GTPases could not be related to the GTP or GDP ligand conformation of the enzyme.

### Potential role of small GTPases in OTA carcinogenicity

OTA genotoxicity is thought to involve mitotic aberrations as a consequence of replication stress (Klotz et al. 2025). Both DNA replication and mitosis are intricate processes tightly regulated by complex networks of protein interactions and cell signaling, which often involve small GTPases as either indirect or direct regulatory switches. Therefore, several hypotheses as to how dysregulation of small GTPases may contribute to biological effects induced by OTA appear plausible.

The Ran GTPase is the key regulator of active nuclear import of proteins with either canonical or non-canonical import signals. Among these proteins are factors required for DNA replication, like core constituents of the replisome but also structuring factors such as histones (Bernardes and Chook 2020). During S-phase, histones must be supplied constantly to enable packaging of nascent DNA and chromatin compaction (Bernardes and Chook 2020). Lack or decrease of histone supply has been shown to slow DNA replication and is thus recognized as a cause of replicative stress and associated DNA damage (Almeida et al. 2018; Mejlvang et al. 2014). Indeed, we previously speculated that decreased histone supply may play a role in the slowing of replication fork progression induced by OTA (Klotz et al. 2025). Inhibition of nuclear import of histones and/or other replications factors via direct interaction of OTA with Ran GTPase as the molecular initiating event may thus provide a mechanistic explanation for replication stress and subsequent events induced by OTA that ultimately promote genomic instability. In addition to its role in mediating nuclear import, Ran is also actively involved in mitosis, particularly during initial spindle assembly and nuclear envelope formation (Chang et al. 2019; Clarke and Zhang 2008; Kalab et al. 1999; Matchett et al. 2014). These functions may provide a potential direct link between the putative OTA target Ran and mitotic aberrations induced by OTA (Czakai et al. 2011). Disruption of the Ran GTP–GDP cycle, for example, has been demonstrated to cause aberrant mitotic figures such as multipolar spindles or defective chromosome alignment (Moore et al. 2002), which is also observed in kidney epithelial cells exposed to OTA (Czakai et al. 2011; Rached et al. 2006). Moreover, the presence of RanGTP with functional GTPase activity has been shown to be required for formation of nuclear envelopes in late telophase along with chromatin decondensation (Clarke and Zhang 2008; Hetzer et al. 2000).

Similarly, several members of the closely related Rab subfamily, which is mainly involved in regulation of vesicular trafficking, have been shown to play a crucial role in nuclear envelope membrane recycling and restructuring of membrane organelles, spindle assembly and chromosome alignment during mitosis (Jimenez et al. 2023; Lanzetti 2012; Miserey-Lenkei and Colombo 2016; Stenmark 2009). Particularly late stage mitotic cytokinesis is driven in part by membrane trafficking (Albertson et al. 2005) orchestrated by Rab and Arf small GTPases (Frémont and Echard 2018). In particular, Rab5 and 11, two Rab GTPases of the endosome pathway involved in nuclear envelope (Lanzetti 2012) and Golgi recycling (Frémont and Echard 2018), have been reported to localize around centrosomes and spindle poles during mitosis (Miserey-Lenkei and Colombo 2016; Serio et al. 2011). Transient knockdown of Rab5 using RNA interference has been shown to cause delayed nuclear envelope breakdown as well as aberrant chromosome alignment during metaphase (Lanzetti 2012; Serio et al. 2011).

Besides its role in mitosis, Rab-mediated vesicular trafficking is essential for maintaining compartment functionality by regulating the transport of proteins and lipids between organelles as well as facilitating endo- and exocytosis processes (Stenmark 2009). Vesicular trafficking between the ER and Golgi apparatus, which is mediated by Rab1- and COP2-coated vesicles (Stenmark 2009), is crucial for protein maturation of membrane proteins, post-translational modifications (glycosylation, sulfation, phosphorylation and proteolytic processing) (Connerly 2010) and proper protein targeting. Interference with either of these important basic cellular processes may therefore disrupt cell homeostasis, potentially contributing to genotoxicity.

Besides membrane restructuring, spindle assembly and chromosome alignment, other critical processes in mitosis are regulated by progressive cytoskeletal dynamics. Faithfull separation of the genome relies on spindle microtubule contraction, while final membrane separation in cytokinesis relies on actomyosin ring constriction. While initially Rho GTPases were only believed to regulate actin dynamics, they are now known to be also involved in the regulation of microtubule dynamics both directly via formins or p21-activated kinase 1 (PAK1) and indirectly via various actin-microtubule filament interfaces and shared regulators (Chircop 2014; Conde and Cáceres 2009; Etienne-Manneville 2013; Li and Gundersen 2008; Pimm and Henty-Ridilla 2021; Wojnacki et al. 2014).

Additionally, Rho GTPases RhoA and Rac1 have been shown to play a role as signal transducers in DNA damage response (DDR) signaling (Cheng et al. 2021; Fritz and Henninger 2015; Magalhaes et al. 2021). We previously speculated that replication-coupled DNA damage induced by OTA may fail to fully activate DDR pathways, which may allow damaged cells to enter into mitosis and thus jeopardize genomic integrity (Klotz et al. 2025). Interference of OTA with Rho GTPase signaling may therefore contribute to its genotoxicity.

Moreover, effects of OTA on cell adhesion and cell communication, including increased tight junction permeability (McLaughlin et al. 2004), downregulation of connexins and decreased gap junction intercell communication (Adler et al. 2009; Mally et al. 2006), as well as decreased cellular adhesion (Mally et al. 2006; Scibelli et al. 2003) may also be linked to potential effects on Rho family GTPases. Actin-associated Rho GTPases RhoA, Rac1, and Cdc42 have been shown to regulate tight junction integrity (Citi et al. 2014; Varadarajan et al. 2022; Zihni and Terry 2015), gap junction communication (Jara et al. 2024; Smyth et al. 2012), and cell/focal adhesion (Burridge and Wennerberg 2004; Morgan et al. 2007; Wojnacki et al. 2014) by controlling actin cytoskeleton dynamics and junctional protein localization.

Finally, Ras subfamily small GTPases are key regulators in proliferation, differentiation and survival signaling pathways and among the most well-known proto-oncogenes, with Ras family proteins being mutated in about 20–30% of all cancers (Kolch et al. 2023; Prior et al. 2020). Interestingly, the majority of oncogenic Ras mutation have been shown to alter the GTP/GDP binding site, which we also identified as the most plausible site of OTA-small GTPase interaction, ultimately compromising GTPase function (Prior et al. 2020). Constitutively active Ras, either by mutation or non-hydrolysable ligand induced stabilization of the GTP conformation, induces persistent mitogenic signaling and accelerates cell cycle progression (Chen et al. 2025; Khan et al. 2019; Punekar et al. 2022; Segeren et al. 2022). The consequential shortening of the G_1_ -phase (Segeren et al. 2022) limits the accumulation of critical components required for faithful DNA synthesis, including histones, nucleotides, and replisome constituents (Primo and Teixeira 2019; Técher et al. 2024). Additionally, hyperactive Ras enables excessive origin licensing via CDC6 overexpression (Di Micco et al. 2006; Técher et al. 2024), as well as transcription-replication conflicts by sustained activation of various transcription factors (Bayona-Feliu and Aguilera 2025; Kotsantis et al. 2016; Primo and Teixeira 2019; Técher et al. 2024). These combined effects constitute key mechanisms of oncogene-induced replication stress, ultimately contributing to genomic instability and tumor formation (Técher et al. 2024).

### Considerations and limitations

Besides the general limitations of shotgun proteomics, such as limited coverage, sequence allocation, and protein identification (Dupree et al. 2020), affinity chromatography introduces additional specific challenges. Most notably, chemical derivatization of the compound is required for immobilization. In this study, masking the carboxyl group via polyamine linker attachment was considered acceptable as the phenylalanine motif appears to be dispensable for OTA toxicity and thus target interaction. However, steric effects from derivatization may still impact binding affinities as the flexible polar linker, while allowing access to buried pockets, may restrict OTA orientations, particularly those where the spacer intrudes into the protein interior. Another limitation of the single-step immobilization chemistry used here is the tendency of carbodiimide-activated *O*-acylisourea intermediates to undergo racemization (El-Faham and Albericio 2011). As a result, immobilized OTA and phenylalanine likely exist as racemic mixtures, preventing stereoselective target screening of 2S-OTA. Relative proteome compositions and protein conformations of protein extracts are strongly affected by lysis and extraction conditions used, which may particularly affect membrane proteins (due to their hydrophobic transmembrane domains collapsing and limited solubilization) and metalloproteins (which can lose metal cofactors under chelating or reducing conditions), potentially hindering affinity capture of these proteins. While allowing weaker target binding, mild (low detergent) conditions during incubation and washing, may also maintain protein–protein interactions and therefore result in a mix of direct binding proteins and their respective interaction partners, which cannot be distinguished by this method.

## Conclusion

In summary, results from the present study identify small GTPases as potential target proteins of the mycotoxin and renal carcinogen OTA using affinity chromatography-based chemoproteomics complemented by in silico simulations. Computational analysis confirmed stable binding interactions with representatives of all small GTPase subfamily GTP-binding domains and implies the potential of ochratoxins to dysregulate GTPase functionality. Simulations also indicate the OTA metabolite OTα as a ligand with higher affinity compared to OTA, independent of GTP/GDP conformations of the GTPases. These findings provide an experimental basis to develop new mechanistic hypotheses that link small GTPases as potential molecular targets of OTA to key events thought to be critical in OTA genotoxicity. However, other OTA-binding proteins identified in this study may also represent mechanistically relevant targets, and their potential role in OTA toxicity remains to be explored.

## Supplementary Information

Below is the link to the electronic supplementary material.

Supplementary file1 (PDF 849 KB)

Supplementary file2 (XLSX 16,444 KB)

## Data Availability

All data supporting the findings of this study are available within the paper and its Supplementary Information. Additional raw data are available from the corresponding author upon reasonable request.

## Abbreviations

AC: Affinity chromatography (also Affinity Capture)
BSA: Bovine serum albumin
DAVID: Database for Annotation, Visualization and Integrated Discovery
DDR: DNA damage response
DMSO: Dimethyl sulfoxide
EDC-HCl: 1-Ethyl-3-(3-dimethylaminopropyl)carbodiimide hydrochloride
EFSA: European food safety authority
GAP: GTPase-activating protein
GDP: Guanosine-5’-diphosphate
GEF: Guanosine triphosphate exchange factor
GO: Gene ontology
GTPase: Guanosine-5’-triphosphate hydrolase
GTP: Guanosine-5’-triphosphate
HK-2: Human kidney cells
MES: 2-(N-morpholino)ethanesulfonic acid
MIE: Molecular initiating event
MOA: Mechanism of action
OTA: Ochratoxin A
PBS: Phosphate-buffered saline
Phe: (l)-phenylalanine
RT: Room temperature
TWI: Tolerable weekly intake
MD: Molecular Dynamics
RMSD: Root Mean Square Deviation
