# Correction: Identification of small GTPases as potential target proteins of the mycotoxin and renal carcinogen ochratoxin A

**DOI:** 10.1007/s00204-025-04225-7

**Published:** 2025-10-25

**Authors:** Johannes Borchers, Florinda Perugino, Andreas Schlosser, Stephanie Lamer, Leonie Lutz, Lorenzo Pedroni, Luca Dellafiora, Angela Mally

**Affiliations:** 1 Department of Toxicology, University of Würzburg, Versbacher Str. 9, 97078 Würzburg, Germany; 2 Rudolf Virchow Center, University of Würzburg, Würzburg, Germany; 3 Department of Food and Drug, University of Parma, Parma, Italy

**Correction: Archives of Toxicology** 10.1007/s00204-025-04189-8

In this article, the first name and family names of the authors were swapped,

Borchers Johannes · Perugino Florinda · Schlosser Andreas · Lamer Stephanie · Lutz Leonie · Pedroni Lorenzo · Dellafiora Luca

The original article has been corrected.

