# Supplementary material for: Identification of small GTPases as potential target proteins of the mycotoxin and renal carcinogen ochratoxin A
Source: Arch Toxicol. 2025 Sep 11;100(1):367–86. doi: 10.1007/s00204-025-04189-8 (PMC12858599; doi:10.1007/s00204-025-04189-8)
Supplement: Supplementary file 1 — Supplementary file1 (PDF 849 KB) [file 204_2025_4189_MOESM1_ESM.pdf]

Identification of small GTPases as potential target proteins of the mycotoxin and renal carcinogen Ochratoxin A

Borchers, Johannes [1]; Perugino, Florinda [3]; Schlosser, Andreas [2]; Lamer, Stephanie [2]; Lutz, Leonie [1]; Pedroni, Lorenzo [3]; Dellafiora, Luca [3]; Mally, Angela [1]

[1]University of Würzburg, Department of Toxicology, Würzburg, Germany
[2]University of Würzburg, Rudolf Virchow Center, Würzburg, Germany
[3]University of Parma, Department of Food and Drug, Parma, Italy

Supplementary Figures 1-4

Supplementary Figure 1

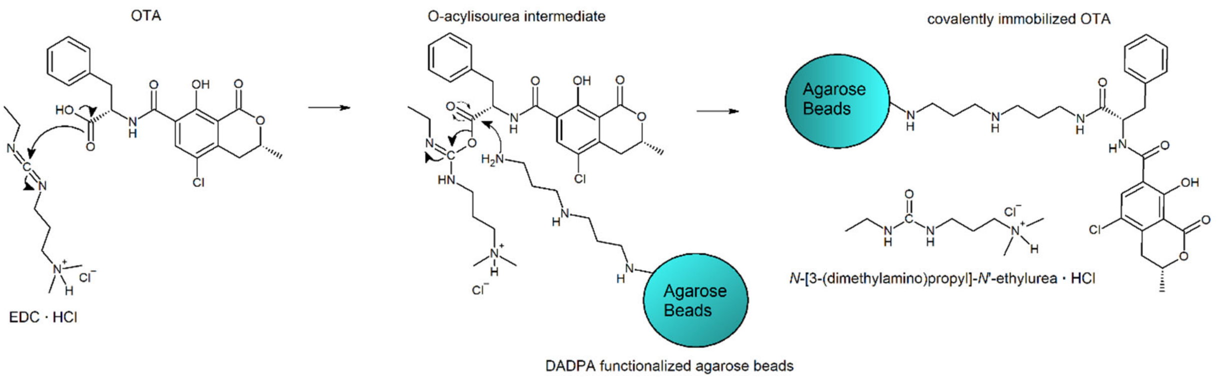

Figure 1: Detailed reaction scheme of the carbodiimide mediated covalent immobilization of OTA on agarose beads. The water-soluble urea derivative side product is removed by repeated washing after coupling incubation. The activated O-acylisourea intermediate is subject to racemization.

Supplementary Figure 2

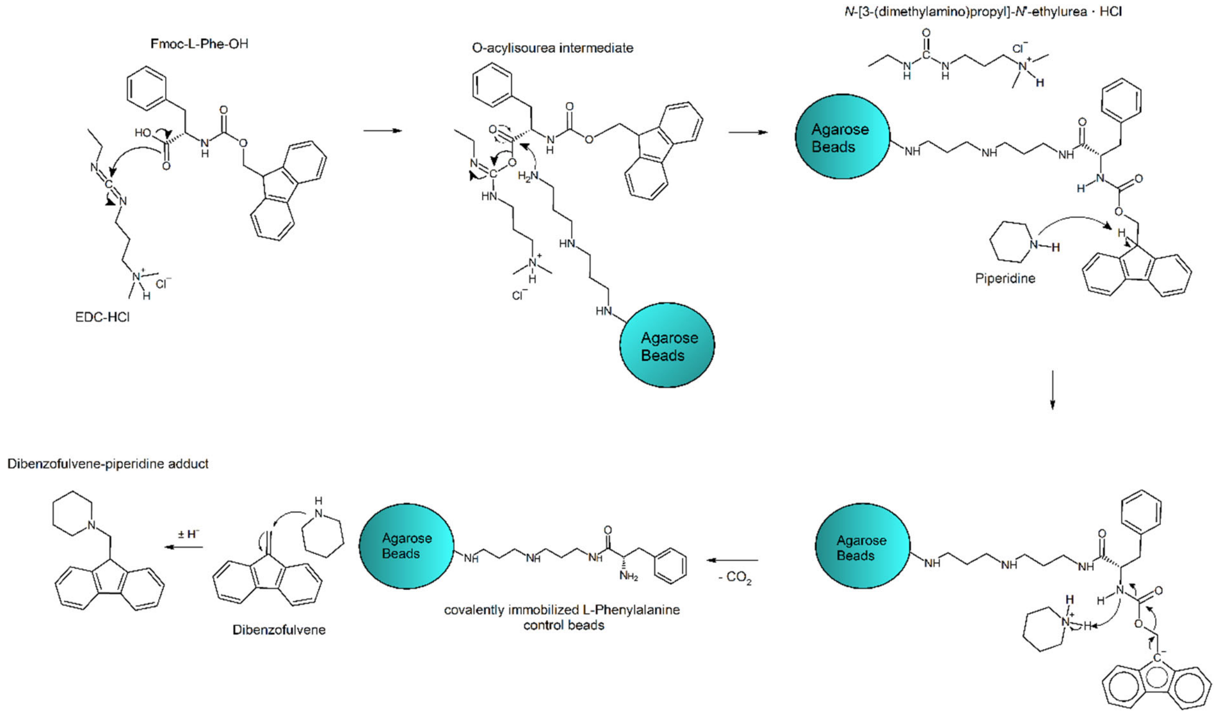

Figure 2: Detailed reaction scheme of the carbodiimide mediated covalent immobilization of Fmoc-L-phenylalanine on agarose beads. The water-soluble urea derivative side product is removed by repeated washing after coupling incubation. The activated O-acylisourea intermediate is subject to racemization. Deprotection of phenylalanine amine is facilitated by base catalyzed cleavage of carbamate. Piperidine is used as a base to allow for scavenging of the dibenzofulvene deprotection product to prevent irreversible adduct formation.

Supplementary Figure 3

A

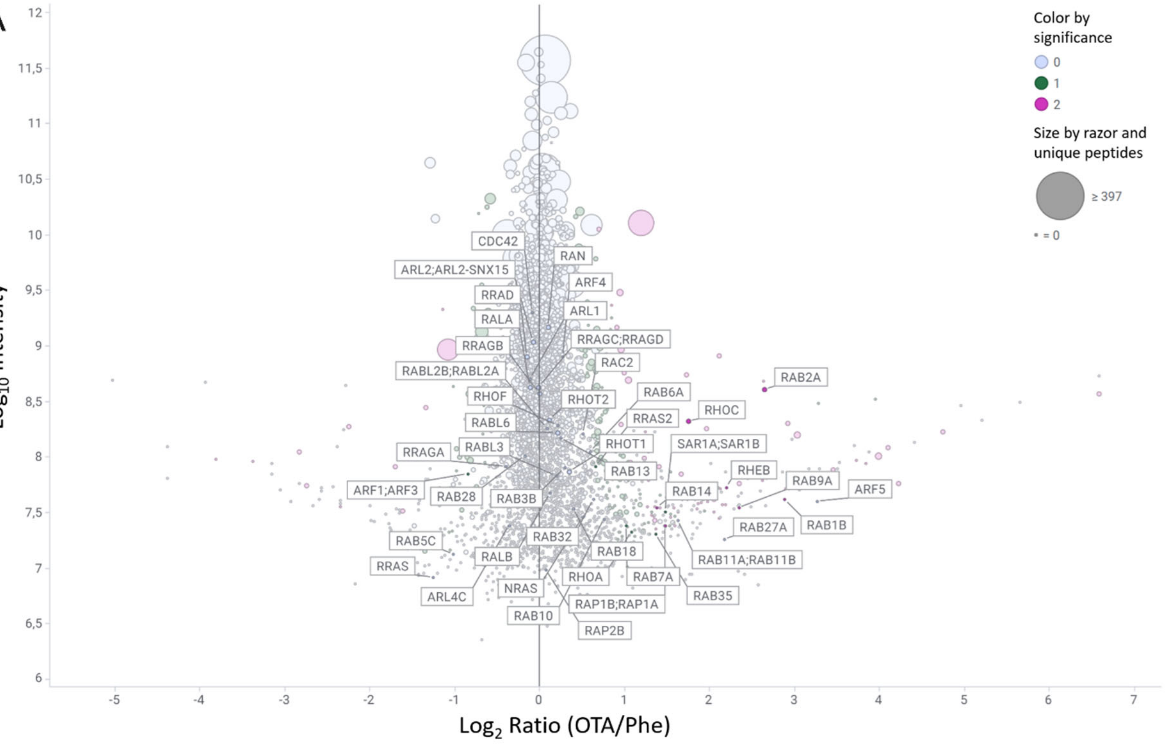

B

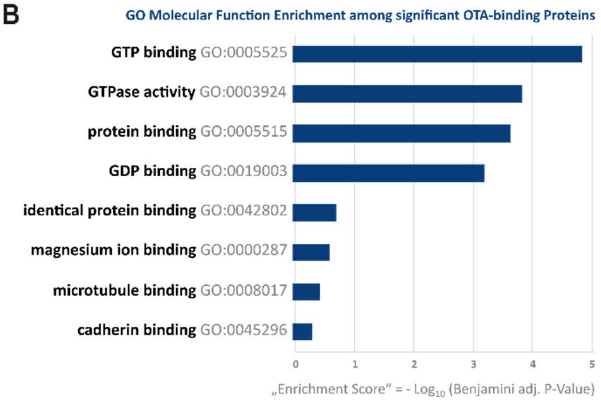

C

Annotation Cluster 1	Enrichment Score: 5.19	Filtered list: Significance 2, Log_2 Ratio> 0 n=80		
**Category**	**Term**	**Count**	**%**	**Benjamini adj. P-Value**
GOTERM MF DIRECT	GO:0005525 **GTP binding**	13	16.25	1.54E-5
INTERPRO	IPR005225: **Small GTP-binding protein domain**	9	11.25	1.18E-4
GOTERM MF DIRECT	GO:0003924 **GTPase activity**	11	13.75	1.49E-4

Figure 3: OTA target protein identification using OTA vs. Phe affinity chromatography of whole cell HK-2 lysates. (A): relative quantification of all identified proteins with intensities and intensity ratios presented as median of 3 independent biological replicates. Proteins marked with their gene name represent Ras superfamily proteins, showing their high prevalence among the significantly OTA enriched (positive Log_2 ratio, purple). (B) Molecular function gene ontology analysis showing significant enrichment of GTPase functionality, G-protein activity and related terms. (C) Functional annotation clustering of molecular function and InterPro category terms revealed GTPases, mostly consisting of Ras superfamily small GTPases, as functional annotation cluster with the highest enrichment score (-Log10 transformed mean of P-values of contained enriched terms) and fold enrichments.

Supplementary Figure 4

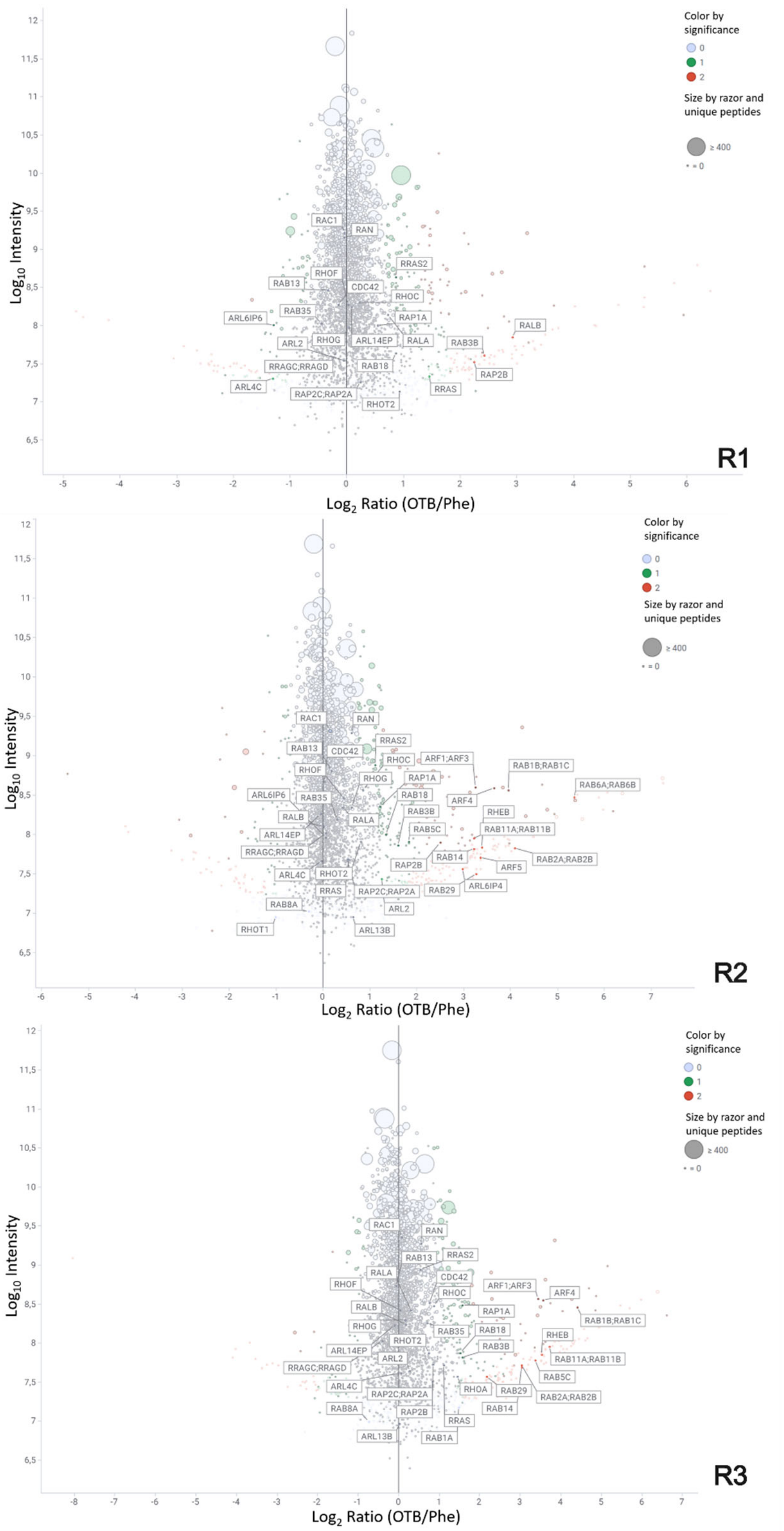

Figure 4: Relative quantification of individual replicates of OTB vs. Phe affinity chromatography from nuclear and organelle enriched extracts. Proteins marked with their gene name represent Ras superfamily proteins, showing their high prevalence among the significantly OTB enriched (positive Log_2 ratio, purple) proteins in replicate two and three (R2, R3). Number of small GTPases identified and OTB enriched was decreased in first replicate resulting in lower number of GTPases significantly enriched in triplicate.
